# Different trajectories of the first-episode psychosis remission in young adults

**DOI:** 10.1192/j.eurpsy.2022.802

**Published:** 2022-09-01

**Authors:** V. Kaleda, D. Tikhonov

**Affiliations:** FSBSI «Mental Health Research Centre», Department Of Youth Psychiatry, Moscow, Russian Federation

**Keywords:** First Episode Psychosis, remission, remission formation, post psychotic personality disorders

## Abstract

**Introduction:**

Analysis of the first-episode psychosis remission, including post-psychotic affective and primary negative symptoms as well as personality changes, is necessary to personalize therapy and rehabilitation.

**Objectives:**

We aimed to identify different trajectories of psychosis remission in young adults.

**Methods:**

First-episode psychosis patients (n=56, mean age 19.8±2.5 years, all males) underwent psychopathological assessment at the stage of remission.

**Results:**

Three trajectories of remission were identified. The thymopathic trajectory (33.93%, 19 patients) was characterized by the gradual increase of subclinical affective symptoms and resulted with a high-quality remission. In 63.61% cases in this group persistent depressed mood was present after a psychotic episode. Some patients (36.84%) became prone to depressive reactions. The pathocharacterological trajectory (39.28%, 22 patients) was characterised by personality changes with increase of existing traits or the development of traits previously not present. Types with an increase of schizoid (14.29%), histrionic (19.64%), and anxiety-hypochondriacal (5.36%) traits were identified. Patients in this group had high- as well as low-quality remission. The destructive trajectory (26.79%, 15 patients) was characterised by residual positive or single negative symptoms. Patients in this subgroup had low-quality remission with poor functioning and signs of treatment resistance.

**Conclusions:**

Analysis of trajectories of the first-episode psychosis remission allowed us to choose the most effective strategy for personalized supportive treatment.

**Disclosure:**

No significant relationships.

